# Refinement and Preliminary Validation of the Technological Competency as Caring in Healthcare Instrument (TCCHI): Psychometric Evaluation of a Concise Three-Factor Model

**DOI:** 10.3390/healthcare14101394

**Published:** 2026-05-19

**Authors:** Risa Yamanaka, Hirokazu Ito, Yoshiyuki Takashima, Krishan Soriano, Kaito Onishi, Feni Betriana, Gil Platon Soriano, Allan Paulo Blaquera, Toshiya Akiyama, Ryuichi Tanioka, Rick Yiu Cho Kwan, Jim-Bhoy Viernes, Misao Miyagawa, Kyoko Osaka, Yuko Yasuhara, Masashi Akaike, Tetsuya Tanioka

**Affiliations:** 1 Graduate School of Health Sciences, Tokushima University, Tokushima 770-8509, Japan; yamanaka0025@gmail.com (R.Y.); ksoriano@spup.edu.ph (K.S.); kai10.onishi10@gmail.com (K.O.); jimviernes@spup.edu.ph (J.-B.V.); 2 Graduate School of Biomedical Sciences, Tokushima University, Tokushima 770-8509, Japan; h.itoh@tokushima-u.ac.jp (H.I.); yasuhara@tokushima-u.ac.jp (Y.Y.); akaike.masashi@tokushima-u.ac.jp (M.A.); 3 School of Medicine, Faculty of Nursing, Nara Medical University, Nara 634-8521, Japan; y-takashima@naramed-u.ac.jp; 4 Center for Biomedical Research, National Research and Innovation Agency (BRIN), Cibinong 16911, Indonesia; feni004@brin.go.id; 5 Department of Nursing, College of Allied Health, National University, Manila 1008, Philippines; gil.p.soriano@gmail.com; 6 School of Nursing and Allied Health Sciences, St. Paul University Philippines, Cagayan 3500, Philippines; ablaquera@spup.edu.ph; 7 Department of Public Health Nursing, Faculty of Health and Welfare, Kawasaki University of Medical Welfare, Kurashiki 701-0193, Japan; a.5.t.2@mw.kawasaki-m.ac.jp; 8 Faculty of Health Sciences, Hiroshima Cosmopolitan University, Hiroshima 731-3166, Japan; tanioka@hcu.ac.jp; 9 School of Nursing, Tung Wah College, Hong Kong 999077, China; rickkwan@twc.edu.hk; 10 School of Health Sciences, Faculty of Medicine, Tokushima University, Tokushima 770-8509, Japan; misaomiyagawa2023@gmail.com; 11 Department of Nursing, Nursing Course of Kochi Medical School, Kochi University, Kochi 783-8505, Japan; osaka@kochi-u.ac.jp

**Keywords:** confirmatory factor analysis, Delphi, healthcare providers, instrument development, interprofessional collaboration, psychometric validation, technological competency as caring

## Abstract

**Background/Objectives**: With the increasing need for interprofessional team-based care, a practical framework is necessary to evaluate the caring competencies of healthcare providers. This study aimed to develop a refined, concise version of the Technological Competency as Caring in Healthcare Instrument (TCCHI) by: (1) reducing the items from the original 38-item Delphi-validated pool through confirmatory factor analysis (CFA) and (2) providing a preliminary assessment of its structural validity and reliability. **Methods**: An online survey was conducted with 528 healthcare professionals across Japan. The CFA process began with the 38 items identified in a previous Delphi study. To optimize model fit and ensure interprofessional applicability, items were systematically refined based on both statistical criteria and theoretical relevance, resulting in a 12-item, three-factor structure. **Results**: The final 12-item model demonstrated an improved and generally acceptable fit: chi-square to degrees of freedom ratio (χ^2^/*df*) = 3.96, comparative fit index (CFI) = 0.947, Tucker–Lewis Index (TLI) = 0.931, and Root Mean Square Error of Approximation (RMSEA) = 0.0749. All factor loadings were statistically significant (*p* < 0.001) and ranged from 0.437 to 0.83. Composite reliability (CR) for the three factors ranged from 0.700 to 0.827, meeting the threshold for internal consistency. While average variance extracted (AVE) values for some factors were below 0.50, the overall model provided a stable and theoretically consistent structure, albeit as a preliminary psychometric refinement. **Conclusions**: This study provides preliminary evidence for the structural validity and reliability of a refined 12-item, three-factor TCCHI. By offering a concise measurement tool aligned with caring theory, the TCCHI has the potential to support interprofessional assessment, education, and professional development in technology-mediated healthcare environments. However, further research is required to address issues of discriminant validity and confirm measurement invariance across different professional groups.

## 1. Introduction

Modern healthcare faces challenges posed by changing disease patterns, greater medical sophistication, and diversification of patient values. Therefore, a shift from the traditional single-profession healthcare delivery system to a team-based, interprofessional approach has become essential [1,2]. As the super-aged society progresses and the number of people with chronic diseases increases, patients’ needs become more complex. This underscores the growing importance of healthcare, nursing care, and welfare professionals working together organically to provide comprehensive and continuous care [3,4,5]. Collaboration among healthcare professionals is effective in promoting patient-centered care and improving the quality of medical services [6,7]. Against this backdrop, the nature of healthcare delivery is rapidly changing. The introduction of advanced medical technologies such as genomic medicine, artificial intelligence (AI)-assisted image diagnosis, robot-assisted surgery, and telemedicine systems is significantly contributing to improved diagnostic accuracy, maximized treatment efficacy, and the efficient utilization of healthcare resources [8,9,10]. Recently, reports have indicated that “digitized inter-team communication in healthcare”, which is driven by advancements in information and communication technology (ICT) and digital health, contributes to improving the quality of care [11]. Thus, the ability to utilize technology appropriately is an essential element for modern healthcare professionals [12,13].

To conceptually bridge the relationship between technology and caring in healthcare practice, this study is grounded in Locsin’s Technological Competency as Caring in Nursing (TCCN) theory. The TCCN theory proposes that technological competency is not merely a technical skill but an expression of caring that enables healthcare professionals to know persons more fully through technological means [14,15,16]. Within increasingly technology-driven healthcare environments, this perspective provides a valuable framework for understanding how technological knowledge and humanistic care can coexist and mutually enhance one another in clinical practice. The theory suggests that technological proficiency facilitates deeper patient engagement rather than replacing human interaction in healthcare settings [16,17].

The essence of healthcare—respecting patients as individuals and building trust relationships—remains unshaken even as technological advances accelerate [17]. Rather, due to information overload and the rise in non-face-to-face consultations, human connections based on empathetic understanding are now more essential than ever to alleviate patient anxiety and encourage active participation in treatment [18,19,20]. Understanding each patient’s individuality, providing appropriate care tailored to their circumstances, and supporting their self-actualization based on their own values and goals are fundamental elements of high-quality healthcare and constitute the ethical responsibility of healthcare providers [21].

Locsin’s TCCN theory [16] is the conceptual foundation that bridges technology and humanity in this high-tech environment [22]. TCCN theory posits that technological competency is not a separate skill from caring but is an expression of caring itself; it is a tool for fully “knowing” the patient [17]. From this perspective, technological competency is understood as the ability to use technology to deepen understanding of patients as whole persons, rather than merely controlling or managing clinical processes. When appropriately integrated into clinical practice, technology can enhance caring by enabling a deeper understanding of patients’ lived experiences, values, and goals, rather than replacing human relationships. Thus, in this study, technology is defined not merely as medical devices or digital systems but as tools that enable healthcare providers to access, interpret, and integrate patient information to better understand the patient and support individualized care.

Currently, numerous competency assessment scales are tailored to specific professions or specialties [23,24,25]. In environments where modern technology has been widely adopted, measures have been developed to evaluate the perspectives and practices of nursing professionals regarding caring in nursing [25,26,27].

However, a significant research gap exists: these existing instruments are predominantly profession-specific and lack the conceptual breadth to evaluate “caring” as a shared, interprofessional competency. In interprofessional team-based care, the absence of a common evaluative framework often leads to unclear role distribution, communication breakdowns, and fragmented professional growth [28,29,30]. While the need for a shared language of caring is evident, no empirically validated, concise instrument exists to assess technological competency as caring across diverse healthcare disciplines. Although our previous Delphi study established the content validity of a 38-item version of the Perceived Technological Competency as Caring in Healthcare Providers Instrument (TCCHI) [31], its internal structural validity has not been tested, and its length presents a practical barrier to routine clinical use.

To address these gaps, this study focuses on two critical needs: First, the requirement for empirical verification of the scale’s structural validity using confirmatory factor analysis (CFA) among an interprofessional cohort. Second, the urgent need for a more parsimonious instrument to reduce respondent burden and facilitate its application in busy, real-world clinical settings.

The primary objective of this study was to develop and validate a concise version of the TCCHI, specifically tailored for use in interprofessional clinical settings. By refining the instrument through confirmatory factor analysis, this study sought to establish a robust and practical framework to evaluate the caring competencies of diverse healthcare providers. Furthermore, we aimed to assess the structural validity and reliability of the shortened instrument to ensure its utility for interprofessional assessment, education, and professional development.

To evaluate the structural validity of the TCCHI, this study tested the following hypotheses: (1) The perceived technological competency as caring among interprofessional healthcare providers is represented by a clear factor structure. (2) The refined, concise version of the TCCHI maintains a stable factor structure that demonstrates acceptable model fit indices. (3) The dimensions of the TCCHI are positively correlated, reflecting an integrated conceptual framework of technological competency as caring.

## 2. Materials and Methods

### 2.1. Study Design

This study employed an observational cross-sectional design, adhering to the Consensus-based Standards for the selection of health status Measurement Instruments (COSMIN) reporting guidelines [32].

### 2.2. Participants and Data Collection

Eligibility criteria included (a) licensed healthcare professionals within one of 13 specified categories, (b) current employment in a clinical or community health setting in Japan, and (c) voluntary informed consent. To ensure that the data reflected contemporary clinical practice, students, retired professionals, and individuals on long-term leave (e.g., maternity or sick leave) were excluded.

The participants were healthcare professionals in Japan who had given their consent and belonged to one of the following professions: physician, pharmacist, registered nurse, practical nurse, physical therapist, occupational therapist, speech and language therapist, radiology technologist, clinical laboratory technologist, social worker, care worker, psychologist, and registered dietitian. Demographic data collected included gender, age, years of experience, and type of affiliated hospital.

Convenience sampling was employed across 26 hospitals in Japan, with 17 hospitals ultimately participating in this study. Of the 587 individuals who accessed the web-based survey via a Quick Response (QR) code and provided consent, 528 valid responses were included after data cleaning. The sample size was justified based on the subject-to-item ratio [33,34]. General recommendations for CFA suggest a minimum subject-to-item ratio of 10:1 or a sample size exceeding 200–300. Given the initial 38 items, the final sample of *n* = 528 provided a ratio of approximately 14:1, ensuring adequate statistical power and stable parameter estimation.

### 2.3. Measures (Technological Competency as Caring in Healthcare Providers Instrument: TCCHI)

The perceived TCCHI served as the primary measurement scale [31]. Developed in a preceding Delphi study, the TCCHI evaluates the caring competency of interprofessional providers by extending Locsin’s [15] TCCN theory to a broader healthcare context.

The initial scale structure comprised 38 items across six hypothesized factors: (1) promoting self-growth and technological learning; (2) building trusting relationships; (3) providing person-centered care through appropriate technology use; (4) enhancing physical and emotional comfort; (5) promoting patient learning and growth; and (6) engaging in ethico-moral practice and patient advocacy. These 38 items were derived from an initial pool of 67 items generated through a modified Delphi process involving interprofessional experts, which established the instrument’s content validity prior to the present psychometric testing [31]. Items were scored on a 7-point Likert scale (1: Strongly disagree, 2: Disagree, 3: Somewhat disagree, 4: Neither agree nor disagree, 5: Somewhat agree, 6: Agree, 7: Strongly agree). The total scores for the initial 38-item scale ranged from 38 to 266, with higher scores representing a higher perceived level of technological competency as caring in interprofessional healthcare practice.

A complete list of the initial 67 items and the 38 items refined through the Delphi process is provided in our previous report [31].

### 2.4. Data Analysis (Confirmatory Factor Analysis and Psychometric Evaluation)

CFA was conducted because the factor structure of the TCCHI had been theoretically specified in the preceding Delphi study and conceptually grounded in Locsin’s TCCN theory [15,16,23]. CFA is widely recommended when researchers aim to test whether empirical data support a hypothesized latent structure derived from theory or prior research [33,34].

#### 2.4.1. Model Fit Evaluation

Prior to the main analysis, data screening was conducted to ensure data quality. We examined the distribution of each item for normality; while many items showed slight non-normality common in Likert scales, the large sample size (*n* > 500) provided robustness for the maximum likelihood estimation used in CFA. Incomplete responses and “straight-lining” (identical responses across all items) were excluded during data cleaning.

The CFA process began with the 38-item, six-factor initial model. The model fit was evaluated using the following consensus-based criteria [35,36,37]:

- Chi-square to degrees of freedom ratio (χ^2^/df): Accepted if <3.0; acceptable if <5.0.
- Root Mean Square Error of Approximation (RMSEA): Accepted if ≤0.08; ≤0.05 indicates good fit.
- Comparative fit index (CFI), Tucker–Lewis Index (TLI): Accepted if ≥0.90 (good fit if ≥0.95).

#### 2.4.2. Scale Optimization and Item Reduction

To ensure objectivity and transparency in the model refinement process, a systematic four-step optimization algorithm was followed. This algorithm outlines the criteria for retaining or removing items when fit indices are inconsistent or factor loadings are suboptimal. This process iteratively balanced statistical rigor with theoretical integrity as follows:

1. **Model Diagnostics:** We conducted a CFA on the 6-factor, 38-item initial model to establish baseline fit indices (χ^2^/df ratio, CFI, TLI, RMSEA, and 90% confidence interval (CI)).
2. **Theoretical Review:** Each candidate item for deletion was reviewed by an interprofessional team. We assessed conceptual redundancy and theoretical necessity based on the TCCN theory, ensuring that the essence of interprofessional caring was preserved.
3. **Iterative Removal:** Items were removed sequentially, maintaining a minimum of three items per factor. Fit indices were re-evaluated after each iteration to ensure the model progressed toward optimal fit.
4. **Final Validation:** The resulting finalized model was confirmed through a re-run of the CFA to ensure stable parameter estimation and improved parsimony.

#### 2.4.3. Statistical Screening and Validity Assessment

Items were evaluated for potential removal based on two primary criteria: (1) low standardized factor loadings (λ < 0.50) [38] and (2) high modification indices (MIs > 10.0) indicating substantial residual correlations [39]. Following the practical guidelines by Hair et al. [38], items with factor loadings between 0.40 and 0.50 were qualitatively re-evaluated by the research team; those deemed theoretically indispensable for representing core concepts were retained [40].

The internal consistency and convergent validity of the final optimized model were evaluated by calculating the composite reliability (CR) and the average variance extracted (AVE) [41]. Reliability was considered acceptable if CR ≥ 0.70. Convergent validity was considered acceptable if AVE ≥ 0.50, or if CR was >0.60 even when AVE was slightly below 0.50 [42]. Furthermore, discriminant validity was assessed using the Fornell–Larcker criterion, which requires the square root of the AVE for each factor to be greater than its correlations with other factors [42].

#### 2.4.4. Statistical Software

Starting from the initial 38-item pool, items were sequentially removed based on factor loadings and modification indices to achieve the most parsimonious model without introducing any external items. Descriptive statistics were used to summarize the participant demographics. Statistical analyses, including CFA, were performed using the Jamovi Statistical Software (version 2.4; The jamovi project, Sydney, Australia) [43], with a statistical significance level set at *p* < 0.05. Furthermore, CR and AVE were calculated in Microsoft Excel 2019 (Microsoft Corp., Redmond, WA, USA) using the factor loadings and error variances obtained from the CFA results.

## 3. Results

### 3.1. Data Screening and Participant Flow

A total of 587 healthcare professionals completed the survey. During data screening, 59 responses were excluded due to missing data on the TCCHI items, resulting in a complete-case analysis. No significant outliers or “straight-lining” patterns were identified that warranted further exclusion. Consequently, the final sample consisted of 528 valid responses, which were used for the subsequent CFA.

### 3.2. Participant Profile

The demographic characteristics of the 528 participants are summarized in Table 1. The sample was predominantly male (64.8%), with the largest age groups being those in their 40 s (29.7%) and 30 s (24.2%). Professional experience spanned a wide range, from one year to over 40 years; notably, 39.1% of the cohort were seasoned professionals with at least 20 years of experience.

In terms of occupation, the participants represented a well-balanced, interprofessional cohort. Registered nurses (28.6%) constituted the largest professional group, followed by physical therapists (12.3%), occupational therapists (8.7%), pharmacists (6.6%), and physicians (5.8%). The sample also included other specialists, such as radiological technologists, social workers, and registered dietitians. Regarding workplace settings, 44.1% of the participants were affiliated with private hospitals, and 37.1% reported involvement in patient care across all clinical departments.

### 3.3. Confirmatory Factor Analysis Results

Following the statistical criteria, items were sequentially removed to optimize the model. The initial model (Model A), consisting of 38 items and 6 factors, which was first verified, did not meet the proposed fit criteria (χ^2^/df = 5.23, CFI = 0.792, TLI = 0.775, RMSEA = 0.0895). In the initial evaluation of Model A, standardized factor loadings below 0.50 were observed for several items, including Q18 (λ = 0.410), Q24 (λ = 0.402), and Q58 (λ = 0.461). Furthermore, high Modification Indices (MIs) indicated substantial cross-loadings and residual correlations. Notably, items such as Q9 (MI = 98.07), Q45 (MI = 86.13), and Q14 (MI = 56.95) demonstrated significant structural redundancy across multiple factors. Following the refinement algorithm, these items were excluded to achieve statistical parsimony.

This iterative reduction process yielded the final 12-item structure (Model B), which satisfied all predefined fit criteria.

### 3.4. Final Model Fit and Psychometric Properties

The optimization process resulted in a final 12-item model (Model B), comprising three factors with four items each (Table 2 and Table 3). Model B yielded the following fit indices: χ^2^/df = 3.960, CFI = 0.947, TLI = 0.931, and RMSEA = 0.0749 (90% CI: 0.0643–0.0859).

#### Final TCCHI Model: Factor Definitions and Conceptual Integration

The refined TCCHI identifies “Technological Competency as Caring” through three empirically derived factors. These factors synthesize the original theoretical concepts to reflect the practical application of technology in interprofessional healthcare.

**Factor 1: Establishing trust and human connection through professional technological mastery** *(Synthesis of former Concept 1: Promoting self-growth and technological learning and Concept 2: Building trust relationships with patients)*.

- **Definition:** This factor focuses on the integration of technical proficiency with the establishment of trusting relationships. It emphasizes that technological mastery is a foundational requirement for effective patient engagement.
- **Description:** A clear understanding of technological mechanisms and their limitations is a prerequisite for providing individualized care. When technology is used with precision, it enhances workflow efficiency and clinical safety, creating a secure environment for building trust. This factor also includes continuous professional reflection as essential for maintaining the integrity of caring practices in technology-mediated settings.

**Factor 2: Person-centered autonomy support in technologically advanced caring** *(Synthesis of former Concept 3: Providing person-centered care through the appropriate use of technology, and Concept 5: Promoting patient learning and growth)*.

- **Definition:** This factor addresses the use of technology to facilitate patient self-determination and autonomous growth aligned with personal values.
- **Description:** This dimension integrates the patient’s goals and aspirations into the care plan. Within this framework, technology serves as a collaborative platform for guidance and empowerment rather than mere information delivery. By prioritizing transparent information sharing, healthcare providers support patients as active partners in their care, facilitating individualized strategies for self-management and informed decision-making.

**Factor 3: Biopsychosocial comfort and ethico-moral advocacy** *(Synthesis of former Concept 4: Enhancing the physical and emotional comfort of patients, and Concept 6: Engaging in ethico-moral practice regarding technology use and patient advocacy)*.

- **Definition:** This factor encompasses the dual responsibility of promoting holistic well-being through technological intervention while safeguarding patient dignity and rights through ethical advocacy.
- **Description:** Utilizing technology to alleviate physical and mental distress is a fundamental aspect of healing. This factor views the pursuit of biopsychosocial well-being as inseparable from ethical advocacy, including the protection of patient privacy. In technologically advanced environments, this dimension focuses on recognizing the unique value of each individual and defending their rights during clinical interactions.

### 3.5. Reliability and Convergent Validity Assessment

Table 4 presents the reliability and validity indices for the optimized three-factor model. The CR values for the three factors ranged from 0.700 to 0.827. The AVE values were 0.377 for Factor 1, 0.545 for Factor 2, and 0.525 for Factor 3. While the AVE for Factor 1 was below 0.500, its CR reached the threshold of 0.700.

Regarding discriminant validity, the square root of the AVE for each factor was 0.616, 0.738, and 0.725, respectively. The inter-factor correlations ranged from 0.868 to 0.932. As shown in Table 4, these correlations exceeded the square root of the AVE for all factors.

### 3.6. The Final Model Structure

**Figure 1:**
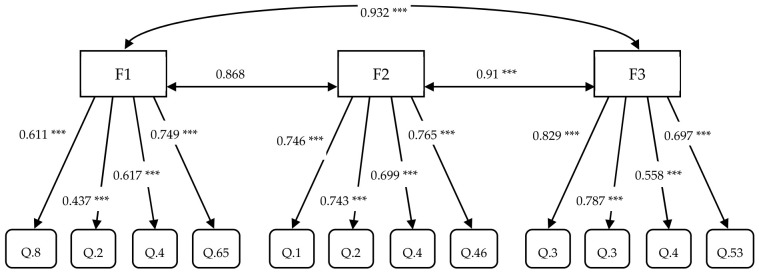
Structure model of the TCCHI based on Model B on Table 2. Note: F: factor. F1: Establishing trust and human connection through professional technological mastery; F2: Person-centered autonomy support in technologically advanced caring; F3: Biopsychosocial comfort and ethico-moral advocacy. Values on the paths represent standardized factor loadings (λ). *** *p* < 0.001. Single-headed arrows indicate the impact of latent factors on observable items. Double-headed arrows indicate the correlations between latent factors. Statistical notation *p* represents the probability value for statistical significance.

Through the iterative optimization process, the initial 38-item model was refined into a 12-item structure (Model B), comprising three factors with four items each. All standardized factor loadings (λ) were statistically significant (*p* < 0.001), with values ranging from 0.437 to 0.829 (Table 5 and Figure 1).

All factor covariances were positive. For this analysis, standardized estimates were used to provide scale-independent coefficients for the evaluation of the factor structure.

## 4. Discussion

### 4.1. Summary of Key Findings and Objective Achievement

This study validated a refined 12-item version of the TCCHI through confirmatory CFA. While the initial 38-item model (Model A) exhibited limitations in structural fit and discriminant validity, the optimized 12-item model (Model B) demonstrated an acceptable fit. Although a χ^2^/df ratio below 3.0 is often ideal, values below 5.0 are widely recognized as acceptable in complex models with substantial sample sizes [37].

These results suggest that this concise 12-item instrument provides a viable means to measure the application of TCCN theory [14] in various interprofessional contexts. This study provides an interprofessional assessment tool grounded in nursing theory, filling a specific need for evidence-based instruments in diverse healthcare settings. These results support the conceptual proposition that technological competency functions as a specialized expression of caring that enhances, rather than replaces, the holistic knowing of persons [14,16].

### 4.2. Discussion of Structural Validity and Factor Composition

The 12-item TCCHI achieves a balance between clinical practicality and statistical rigor. The finding that factor loadings reached statistical significance (*p* < 0.001) with values of λ = 0.437 indicates that each item adequately contributes to its respective latent factor. While a threshold of 0.50 is a common rule of thumb, the inclusion of item Q24 (λ = 0.437) was maintained due to its critical theoretical role in the factor 1. The retention of this item represents an intentional, theory-informed compromise to ensure the conceptual integrity of the instrument. Q24 specifically addresses the evaluative function of technology in understanding patient conditions, a cornerstone of Locsin’s theory, and its exclusion would have diminished the scale’s theoretical alignment.

Furthermore, the transition from the complex six-factor 38-item model to the streamlined three-factor 12-item model significantly reduced the MIs, which were exceptionally high in Model A (exceeding 90.0). This suggests that the iterative optimization process effectively eliminated conceptual redundancies, resulting in a more parsimonious and stable instrument suitable for rapid clinical assessment.

#### 4.2.1. Factor 1. Establishing Trust and Human Connection Through Professional Technological Mastery

This factor underscores that proficient use of technology is essential for providing individualized care rather than focusing solely on patient data [14]. The integration of building trust (Q8), recognizing the patient’s irreplaceability (Q47), and continuous reflection (Q65) underscores that “technological knowing” manifests through relational engagement rather than mere mechanical skill. Interestingly, certain theoretically essential items regarding technology selection (Q18, Q58) did not reach statistical retention. This may be attributed to the diverse nature of our interprofessional sample, whose varied perceptions and daily utilization of “technology” (ranging from specialized medical devices to information management systems) likely introduced statistical variance. This suggests that while technological competency is a shared necessity in modern healthcare, future interprofessional assessments may require nuanced definitions or supplementary instructions that align with the specific technological contexts of different professional roles.

Precise technology use enhances workflow efficiency and clinical safety, fostering a secure environment for building robust, trusting relationships [14,41,42]. This process is sustained by continuous professional reflection, which is essential for the lifelong professional development of healthcare providers and the creation of adaptive learning environments [44,45,46]. The findings suggest that the delivery of specialized care is fundamentally grounded in these trusting relationships. By bridging technical proficiency with compassionate human connection, Factor 1 provides a robust framework for evaluating caring practices within interprofessional teams [47].

#### 4.2.2. Factor 2. Person-Centered Autonomy Support in Technologically Advanced Caring

Factor 2 emphasizes the intentional application of technology to support patient autonomy and self-determination, a core challenge in modern global healthcare. This dimension reaffirms the provider’s role in integrating the patient’s holistic hopes (Q13) and individualized care plans (Q25) into a clinical landscape where technological data can often overshadow human aspirations. The items Q40. providing detailed explanations for self-determination, and Q46. respecting self-determination in support, focus on facilitating self-determination through detailed explanations, transitioning technology from a mere instrumental resource to a relational means of “fully knowing” the person [14,16].

Consistent with TCCN theory, knowing persons occurs through the dynamic processes of technological knowing, designing, and participative engagement [16]. By integrating an understanding of technology with the intentional act of “knowing patient [16]”, this factor underscores that mutual understanding between patients and providers forms the foundation for shared decision-making. Recent evidence highlights that collaborative strategies are essential not only for patient safety but also for fostering a partnership where patients perceive healthcare professionals as reliable, shared decision-makers [48]. When interprofessional teams align their technical expertise with a respect for patient preferences, they reinforce a consistent and secure environment for holistic healing [19,49,50,51].

Furthermore, these items capture a dynamic process in which technology-assisted visualizations and monitoring tools are used not for control, but to enhance health literacy and patient autonomy [52]. By intentionally selecting technology based on individual needs, interprofessional teams ensure that technological advancements preserve the “human touch” instead of diluting the patient’s humanity [53,54]. Such caring behaviors enable patients to improve self-control and achieve self-actualization even within complex, high-tech environments [55]. When interprofessional team members recognize the importance of understanding the patient as a whole and translate this knowledge into clinical practice, their collective expertise enhances the quality of care. This shared approach ensures that technical proficiency is aligned with the patient’s perspective. By fostering a unified strategy grounded in individualized understanding, the interprofessional team can deliver more consistent, safe, and person-centered healthcare services.

#### 4.2.3. Factor 3. Biopsychosocial Comfort and Ethico-Moral Advocacy

This factor bridges universal person-centered values with the rigorous demands of clinical quality [56]. By integrating the promotion of physical and mental comfort (Q35) with the restoration of the patient’s personhood (Q36), this factor evaluates how healthcare providers maintain the “human touch” within high-tech environments. As noted by Borycki and Kushniruk [54], technology should serve as a tool for quality improvement and safety; however, Factor 3 moves beyond technical safety to assess the shared value-judgment criteria required when technology is utilized across multiple professions [57].

A significant finding in this dimension is the strong emphasis on ethical reflection (Q49) and advocacy for patients unable to express their will (Q53). As highlighted in the expert feedback, these items suggest that healthcare providers perceive ethico-moral integrity not as an elective skill, but as the fundamental basis for all clinical interactions. This reflects an “ethical action cycle” [58] where sensitivity and moral awareness translate into observable advocacy-oriented behaviors.

In the evolving digital health landscape, ethical competence constitutes a vital component of caring [59,60,61,62]. These items underscore the necessity of moral intentionality to safeguard patient dignity, echoing Locsin’s warning against reducing persons to digitized data [14]. Furthermore, the inclusion of advocacy reflects the essential ethical competence [63] required of interprofessional teams to ensure that technology complements, rather than obscures, the patient’s humanity. Thus, Factor 3 establishes the TCCHI as a vital evaluation tool for upholding patient dignity in accordance with international ethical codes [64,65].

### 4.3. Reliability and the Challenge of Discriminant Validity

To establish discriminant validity, the square root of the AVE should ideally exceed the corresponding inter-factor correlations [42]. In this study, the shortened 12-item TCCHI demonstrated acceptable internal consistency with CR values of 0.70 or higher. However, we acknowledge that the AVE for Factor 1 (0.377) remained below the 0.50 threshold. Therefore, the convergent validity of this factor should be interpreted with caution as a preliminary finding, reflecting an intentional, theory-informed compromise to maintain the conceptual integrity of the instrument. While some psychometric literature suggests that a factor may still be acceptable if its CR is above 0.60 despite a lower AVE [42], we exercise caution in interpreting these results as definitive evidence of convergent validity.

The high correlations observed among the factors (ranging from 0.868 to 0.932) reflect substantial conceptual overlap. Such strong associations may arise when measurement items capture dimensions that are highly integrated within the latent structure of “knowing persons as caring” [14,16,66]. While these results do not fully satisfy the strict statistical criteria for discriminant validity, they may reflect the practical reality of healthcare, where technological learning and trust-building are perceived as inseparable professional activities. Given the persistent high correlations, we acknowledge that the distinctiveness of these factors requires further empirical scrutiny, and future research should investigate whether a higher-order construct better represents this conceptual synergy [59].

### 4.4. Implications

The development of the 12-item TCCHI followed a process of balancing statistical requirements with theoretical integrity. While eliminating items based solely on statistical criteria can sometimes affect content validity [34], our refinement prioritized identifying a shared “evaluative language” that transcends specific professional roles. The primary purpose of the TCCHI was not to evaluate technical expertise in detail, but rather to assess a cross-disciplinary expression of technological competency as caring applicable to medicine, nursing, rehabilitation, and social welfare.

Consequently, items were selected based on their potential for mutual understanding and applicability across professional boundaries. Items that were heavily dependent on specific professional tasks or might be interpreted differently across roles were carefully reviewed for integration or exclusion. This process was not intended to diminish the depth of caring; rather, it represents an effort to focus on the common core of caring shared within interprofessional teams.

The final 12-item TCCHI aims to capture the core concepts of TCCN by emphasizing the understanding of patients as whole persons and the use of technology as a specialized means of knowing persons [16]. These items focus on professional attitudes and judgments that appear to be applicable across various disciplines. This streamlined structure may facilitate the TCCHI’s role as a common assessment framework within interprofessional teams, potentially enhancing its practical utility in diverse healthcare settings.

Future research is warranted to investigate a higher-order construct that integrates these dimensions to better reflect this conceptual synergy [66].

### 4.5. Study Limitations and Future Research

This study has several limitations that warrant caution in interpreting the findings.

**Sample Composition and Generalizability:** The number of participants varied across professional categories. While this distribution reflects the workforce composition in hospital-based settings—where registered nurses and rehabilitation professionals play central roles in direct care—this imbalance should be considered when generalizing the findings. Although the inclusion of diverse professionals offers initial insights, the sample does not provide a perfectly balanced representation of all healthcare disciplines. Future research should evaluate measurement invariance across different professional groups (e.g., nurses vs. physicians) to confirm whether the factor structure operates consistently across diverse healthcare roles.

**Sampling and Context:** The use of convenience sampling in Japan may limit the generalizability of the results to international healthcare systems with different technological infrastructures and cultural contexts.

**Study Design:** The cross-sectional design precludes the assessment of test–retest reliability and longitudinal stability.

**Model Validation:** Although the optimized 12-item model demonstrated an improved fit, split-sample cross-validation or independent replication was not performed. Consequently, the potential for model overfitting remains a concern, and the current factor structure should be considered a foundational framework requiring further independent validation.

**Clinical Setting Specificity:** The sample included limited representation from specialized units that frequently utilize advanced life-support technologies, such as intensive care or operating rooms. As the perception of technological competency may differ in these high-acuity environments, the current findings may primarily reflect perspectives in general clinical settings.

**Psychometric Properties:** This study primarily focused on structural validity and internal consistency. Other properties, such as criterion-related and predictive validity, were not examined. Future research should investigate whether TCCHI scores are associated with clinical outcomes, such as the quality of interprofessional collaboration and patient-reported experiences, to establish the instrument’s practical utility across various settings.

## 5. Conclusions

This study performed a refinement of the TCCHI into a concise 12-item, three-factor structure and provided initial evidence for its structural validity and reliability. The findings suggest that the instrument offers a promising and theoretically grounded tool for assessing technological competency as caring among multidisciplinary healthcare professionals. However, given the high inter-factor correlations and the current lack of measurement invariance testing across various professions, this finalized model should be regarded as a preliminary framework. By offering a concise measurement scale aligned with TCCN theory, the TCCHI has the potential to support interprofessional education and quality improvement in technology-mediated healthcare environments, provided that further validation studies confirm its psychometric stability across diverse clinical settings.

## Figures and Tables

**Table 1:** Demographic Characteristics (*n* = 528).

Characteristics	Category	*n*	%
Gender	Male	342	64.8
	Female	180	34.1
	Other	0	0
	No response	6	1.1
Age	20–24 years	35	6.6
	25–29 years	71	13.4
	30–39 years	128	24.2
	40–49 years	157	29.7
	50–59 years	100	19.0
	60–69 years	32	6.1
	70 years or older	5	0.9
Years of professional experience in healthcare	1–4 years	86	16.3
	5–9 years	91	17.2
	10–14 years	75	14.2
	15–19 years	70	13.3
	20–29 years	116	22.0
	30–39 years	69	13.1
	40 years or more	21	4.0
Highest educational background in healthcare professions	Nursing high school (vocational program)	14	2.7
	Professional training college	216	40.9
	Junior college	44	8.3
	University	199	37.7
	Graduate school	55	10.4
Professional characteristics of occupation	Physician	31	5.8
	Pharmacist	35	6.6
	Registered nurse	151	28.6
	Practical nurse	10	1.9
	Physical therapist	65	12.3
	Occupational therapist	46	8.7
	Speech-language-hearing therapist	17	3.2
	Radiology technologist	39	7.4
	Clinical laboratory technologist	30	5.7
	Social worker	36	6.8
	Care worker	11	2.1
	Psychologist	13	2.5
	Registered dietitian	44	8.3
Type of hospital workplace	National university hospital	94	17.8
	National hospital organization	26	4.9
	Public hospital (municipal)	38	7.2
	Private hospital	233	44.1
	Other	137	26.0
Clinical department of patients they are involved with	All departments	196	37.1
	Surgical departments	33	6.3
	Medical departments	103	19.5
	Intensive care units (ER, ICU, OR)	24	4.6
	Pediatrics	8	1.5
	Obstetrics and gynecology	6	1.1
	Psychiatry	88	16.7
	Other	70	13.3

**Note**: ER: emergency room, ICU: intensive care unit, OR: operating room.

**Table 2:** Results of Confirmatory Factor Analysis for the Relationship between Two-Type Model Fit Index of TCCHI.

Model	χ^2^	df	χ^2^/df	CFI	TLI	RMSEA	90% CI	
							LL	UL
A	3401	650	5.23	0.792	0.775	0.0895	0.0866	0.0925
B	202	51	3.960	0.947	0.931	0.0749	0.06430	0.0859

**Note**: Structural equation modeling was used for the analysis. χ^2^ = Chi-square; df = degrees of freedom; χ^2^/df = Chi-square to degrees of freedom ratio; CFI = comparative fit index; TLI = tucker–lewis index; RMSEA = root mean square error of approximation; 90% CI = confidence interval; LL = lower limit; UL = upper limit; TCCHI = technological competency as caring in healthcare providers instrument. **Model A (38 items)** was derived from the previous Delphi study but failed to meet fit criteria due to low factor loadings (λ< 0.50 for Q18, Q24, and Q58) and high modification indices (MIs > 50.0 for Q9, Q14, and Q45). **Model B (12 items)** was developed by systematically excluding these problematic items through the iterative refinement process described in Section 2 to achieve optimal statistical fit and clinical parsimony.

**Table 3:** Item Composition by Factor in the Initial (Model A) and Optimized (Model B) TCCHI Models.

Model A (Initial: 6 Factors)		Model B (Final: 3 Factors)	
Factors and Descriptions	Items	Synthesized Factors	Final Items
Factor 1. Promoting self-growth and technological learning	6	**Factor 1. Establishing trust and human connection through professional technological mastery**	4
Factor 2. Building trusting relationships with patients	7	Synthesis of former F1 & F2: Q8, Q24, Q47, and Q65.	
Factor 3. Providing person-centered care through the appropriate use of technology	6	**Factor 2. Person-centered autonomy support in technologically advanced caring**	4
Factor 5. Promoting patient learning and growth	6	Synthesis of former F3 & F5: Q13, Q25, Q40, and Q46.	
Factor 4. Enhancing the physical and emotional comfort of patients	6	**Factor 3. Biopsychosocial comfort and ethico-moral advocacy**	4
Factor 6. Engaging in ethico-moral practice regarding technology use and patient advocacy	7	Synthesis of former F4 & F6: Q35, Q36, Q49, and Q53.	
Total Items	38		12

**Note**: Model A consisted of 38 items derived from the initial 67-item pool developed in the previous Delphi study. Model B is the final 12-item scale optimized through a systematic refinement process. To ensure superior statistical fit and clinical relevance for interprofessional healthcare providers, items were selected and synthesized based on the decision algorithm, resulting in a robust 3-factor structure.

**Table 4:** Reliability and Convergent Validity Assessment Results of the Final 12-item TCCHI Model B.

Factor	Factor Name	AVE	CR	F1	F2	F3
F1	Establishing trust and human connection through professional technological mastery	0.377	0.700	(0.616)		
F2	Person-centered autonomy support in technologically advanced caring	0.545	0.827	0.868	(0.738)	
F3	Biopsychosocial comfort and ethico-moral advocacy	0.525	0.814	0.932	0.91	(0.725)

**Note**: F: factor; AVE: average variance extracted; CR: composite reliability. Diagonal values in parentheses represent the square root of the AVE. Values below the diagonal represent inter-factor correlations (standardized estimates). Following Fornell and Larcker [42], convergent validity is considered adequate if CR > 0.60 even when AVE is <0.50.

**Table 5:** Confirmatory Factor Analysis Results for the 12-item TCCHI.

Factors and Items	UE	S.E.	Z-Value	*p*-Value	Standardized (λ)
**Factor 1: Establishing trust and human connection through professional technological mastery**					
Q8. Acting in a way that builds trust	0.508	0.0352	14.41	<0.001	0.611
Q24. Technology is useful for correctly assessing a patient’s condition.	0.539	0.0556	9.69	<0.001	0.437
Q47. Recognizing the patient as an irreplaceable individual	0.541	0.037	14.60	<0.001	0.617
Q65. Continuous reflection on the caring process	0.726	0.0388	18.71	<0.001	0.749
**Factor 2: Person-centered autonomy support in technologically advanced caring**					
Q13. Supporting hopes and dreams	0.821	0.043	19.11	<0.001	0.746
Q25. Designing individualized care plans	0.864	0.0457	18.92	<0.001	0.743
Q40. Providing detailed explanations for self-determination	0.665	0.0381	17.48	<0.001	0.699
Q46. Respecting self-determination in support	0.735	0.0371	19.81	<0.001	0.765
**Factor 3: Biopsychosocial comfort and ethico-moral advocacy**					
Q35. Promoting physical and mental comfort	0.768	0.0346	22.22	<0.001	0.829
Q36. Aiming to restore the patient’s personhood	0.797	0.0387	20.62	<0.001	0.787
Q49. Thinking about ethical issues in healthcare	0.615	0.0465	13.23	<0.001	0.558
Q53. Advocating for patients who cannot express their will	0.728	0.0414	17.58	<0.001	0.697

**Note**: UE: Unstandardized Estimate; S.E.: Standard Error; (λ): Standardized Factor Loading. All factor loadings are statistically significant at *p* < 0.001.

## Data Availability

The data presented in this study are available upon request from the corresponding author. These data are not publicly available due to privacy restrictions.

## Abbreviations

The following abbreviations are used in this manuscript:

TCCHI	Technological Competency as Caring in Healthcare Providers Instrument
AI	Artificial Intelligence
CFA	Confirmatory Factor Analysis
χ^2^/df	chi-square to degrees of freedom ratio
CFI	Comparative Fit Index
TLI	Tucker–Lewis Index
RMSEA	Root Mean Square Error of Approximation
ICT	Information and Communication Technology
TCCN	Technological Competency as Caring in Nursing
COSMIN	COnsensus-based Standards for the selection of health status Measurement Instruments
QR	Quick Response
MIs	Modification Indices
AVE	Average Variance Extracted
ER	Emergency Room
ICU	Intensive Care Unit
OR	Operating Room
CI	Confidence Interval
LL	Lower Limit
UL	Upper Limit
QOL	Quality of Life
SE	Standard Error
I-CVI	Item-level Content Validity Index
ICC	Intraclass Correlation Coefficient
